# Juvenile idiopathic arthritis associated with a mutation in *GATA3*

**DOI:** 10.1186/s13075-019-1946-3

**Published:** 2019-06-25

**Authors:** Anna E. Patrick, Wei Wang, Elly Brokamp, Thomas Brent Graham, Thomas M. Aune, Jessica B. Duis

**Affiliations:** 1 Division of Rheumatology, Department of Pediatrics, Vanderbilt University Medical Center, 1211 Medical Center Drive, Nashville, TN 37232 USA; 2 GeneDx, Inc, 207 Perry Parkway, Gaithersburg, MD 20877 USA; 3 Division of Medical Genetics and Genomic Medicine, Department of Pediatrics, Vanderbilt University Medical Center, 1211 Medical Center Drive, Nashville, TN 37232 USA; 4 Division of Rheumatology and Immunology, Department of Medicine, Vanderbilt University Medical Center, 1211 Medical Center Drive, Nashville, TN 37232 USA

**Keywords:** GATA3, Autoimmune arthritis, Juvenile idiopathic arthritis, T cell

## Abstract

**Background:**

GATA3 is a transcription factor that is important during development and plays a role in differentiation and activity of immune cells, particularly T cells. Abnormal T cell function is found in autoimmune arthritis. We present the first known case of autoimmune arthritis associated with a novel *GATA3* mutation.

**Methods:**

Whole exome sequencing of the proband was performed on a clinical basis. Peripheral blood mononuclear cells (PBMCs) were collected from the proband, healthy sibling, and parent. cDNA prepared from RNA was analyzed with polymerase chain reaction and Sanger sequencing. Intracellular proteins were assessed by immunoblot of PBMC homogenates. GATA3 in vitro activity was measured in HeLa cell cultures expressing a mammalian expression vector containing GATA3 or mutants generated by site-directed mutagenesis. GATA3 transcriptional activity was examined using a luciferase reporter assay system. T helper cell ex vivo function was evaluated by stimulating PBMCs to differentiate into effector T cells along Th0, Th1, Th2, and Th17 lineages, and re-stimulating effector cells to secrete cytokines. Cytokine production was measured by enzyme-linked immunosorbent assay.

**Results:**

The proband is the first known case of autoimmune arthritis associated with a mutation in *GATA3*. The proband M401VfsX106 protein is expressed and has a dominant negative function on GATA3 transcriptional activity. The proband PBMCs have markedly increased differentiation along the Th1 and Th17 pathways, with decreased differentiation along the Th2 pathway. Unexpectedly, Th0 cells from the proband express high levels of IFNγ.

**Conclusions:**

Our research presents the first known case of autoimmune arthritis associated with a mutation in *GATA3*. This work expands the phenotypic spectrum of *GATA3* mutations. It reveals the novel insight that decreased and altered GATA3 activity coincides with autoimmune arthritis. This work suggests that modulation of GATA3 may be a therapeutic approach for patients with autoimmune arthritis.

**Electronic supplementary material:**

The online version of this article (10.1186/s13075-019-1946-3) contains supplementary material, which is available to authorized users.

## Background

GATA3 is essential for early thymocyte and mature peripheral T cell development [1]. In T cells, GATA3 is the master transcription factor driving naïve T cell differentiation to effector T helper 2 (Th2) cells. Conversely, GATA3 suppresses Th1 and Th17 cell differentiation. In Th1 cells, this occurs by a counter-regulatory interaction with the Th1 master transcription factor, T-bet [2]. Th1, Th2, and Th17 cells are important for clearance of intracellular, helminth, and extracellular and fungal pathogens respectively [3]. An increase in Th2 cell activity associates with atopic disease, whereas increases in Th1 and Th17 cell activity associate with autoimmune and inflammatory conditions.

Autoimmune diseases, including rheumatoid arthritis (RA), are linked to an increase in Th1 and Th17 activity [4]. In both children and adults with autoimmune arthritis, Th1 and Th17 cells are present in inflamed synovium [5–7]. In murine models, Th2 cells have a role in suppressing inflammatory arthritis [8]. Moreover, *GATA3* is a susceptibility locus associated with rheumatoid arthritis, though mechanistic detail is lacking [9].

Patients with rare defects in the gene encoding *GATA3* have hypoparathyroidism, sensorineural deafness, and renal disease (HDR) (MIM 146255) resulting from decreased GATA3 function [10]. In one case report, a pediatric HDR patient also developed type I diabetes [11]. The studied *GATA3* mutations result in loss of function [12, 13], with one missense mutant in a DNA-binding domain causing a dominant negative [14]. Stimulation of T cells from HDR patients suggests an increase in Th1 and a reduction in Th2 phenotypes, with an overall Th1-dominated immunity [15].

Whole exome sequencing (WES) is quickly becoming a standard of care in patients with complex phenotypes, particularly when features suggest the possibility of two different genetic disorders. Studies suggest improved diagnostic rates and cost-effectiveness by ending the diagnostic odyssey in positive cases [16–18]. We performed WES of our proband who has congenital ptosis, strabismus, bilateral sensorineural hearing loss, bilateral choanal atresia, endocrinopathies, renal cysts, and psoriatic juvenile idiopathic arthritis (JIA). We identified a de novo heterozygous mutation in *GATA3* that causes a frameshift with stop-loss and elongation of the C-terminus and has a dominant negative mechanism.

## Methods

### Genetic studies

Trio whole exome sequencing was performed on a clinical basis. Using genomic DNA from the proband and parents, the exonic regions and flanking splice junctions of the genome were captured using the IDT xGen Exome Research Panel v1.0. Massively parallel (NextGen) sequencing was done on an Illumina system with 100 bp or greater paired-end reads. Mean coverage of captured regions was ~ 102× per sample, with > 98% having a depth of coverage of at least 10×. Reads were aligned to human genome build GRCh37/UCSC hg19 and analyzed for sequence variants using a custom-developed analysis tool. Additional sequencing technology and variant interpretation protocol has been previously described [19]. The general assertion criteria for variant classification are publicly available on the GeneDx ClinVar submission page (http://www.ncbi.nlm.nih.gov/clinvar/submitters/26957/).

### GATA3 expression

Peripheral blood mononuclear cells (PBMCs) were collected from the proband and healthy 2-year-old brother using density separation. RNA was purified and complementary DNA (cDNA) synthesized. Segments of the *GATA3* gene containing the mutation were amplified using polymerase chain reaction (PCR) from cDNA. Sanger sequencing was performed. Immunoblotting was performed with PBMC homogenates and rabbit monoclonal antibody against GATA3 (Abcam ab214804) and HRP-conjugated mouse monoclonal antibody against actin (Promega). Quantitative real-time PCR was used to assess expressed mRNAs using SYBR green master mix (Applied Biosystems).

### GATA3 in vitro activity

HeLa cells were transfected with a vector containing the luciferase gene driven by GATA binding sites (pGL3 GATA Luc, Addgene) [20] and constructs containing wildtype (RSV/hGATA3, Addgene) [21] and/or mutant GATA3. GATA3 mutants were generated by site-directed mutagenesis and included the proband mutation M401VfsX106, a M401X truncation, and three HDR-associated mutants, a G248X truncation, a C321S missense mutation, and H400HfsX107. G248X removes both critical sites for DNA binding. C321S mutates a critical position in the DNA-binding domain and has been characterized as a dominant-negative mutant [14]. H400HfsX107 results in frame shift at position 400 with a 107 amino acid elongation [11]. After transfection, HeLa cells were lysed and luciferase activity determined using a Luciferase Assay System (Promega).

### GATA3 ex vivo function in Th cells

Functional studies were performed by assessing the potential of the proband and sibling T cells to differentiate into effector T cells along Th0, Th1, Th2, and Th17 lineages and secrete respective cytokines upon re-stimulation. Proband PBMCs were obtained at two separate timepoints, 9 months and 12 months, after diagnosis of JIA. PBMCs from the sibling were obtained on the 12-month timepoint. PBMCs from the 9-month collection were assessed immediately after collection. PBMCs from the 12-month collection were frozen in fetal bovine serum with 10% DMSO and then thawed immediately before assessment. We use well-defined tissue culture model protocols for Th cell differentiation and cytokine secretion, referred to as the Th differentiation assay [22]. In this assay, PBMCs were induced to proliferate on plates precoated with anti-CD3 (10 μg/mL, OKT3 clone, prepared in Aune laboratory) with the addition of soluble anti-CD28 (1 μg/mL, Biolegend). Cytokines were added to drive differentiation along Th differentiation pathways for 5–7 days, Th1: interleukin (IL)-12 (10 ng/mL), Th2: IL-4 (10 ng/mL), or Th17: IL-6 (50 ng/mL), IL-1β (20 ng/mL), TGF-β1 (2 ng/mL), IL-23 (20 ng/mL), IL-21 (100 ng/mL), anti-interferon (IFN)γ (10 μg/mL). IL-12, IL-4, IL-6, and IL-1β were purchased from BD Biosciences. Anti-IFNγ was purchased from BD Pharminogen. IL-1β, TGF-β1, IL-23, and IL-21 were purchased from R&D Systems. Differentiated Th cultures were harvested and re-stimulated on plates precoated with anti-CD3. Cultures were collected after 2 days and analyzed for levels of these cytokines by enzyme-linked immunosorbent assay (ELISA), Th1: IFNγ, Th2: IL-5 and IL-13, Th17: IL-17A, Th0: IFNγ. IFNγ and IL-5 ELISA kits were purchased from BD Biosciences. The IL-13 and IL-17A ELISA kits were purchased from ThermoFisher Scientific.

### Statistical analysis

All applied statistical tests were two-tailed unpaired *t* test with Welch’s correction. *P* < 0.05 was considered significant.

## Results

### Case report

Our proband presented as a 3 year-old boy with developmental delay, choanal atresia, hypoparathyroidism, hypothyroidism, ptosis, renal cysts, sensorineural hearing loss, psoriasis, and polyarthritis that was diagnosed as JIA, psoriatic subtype. At onset, he had arthritis in bilateral knees and ankles, left hip, multiple metacarpals, and dactylitis in one toe (Additional file 1: Figure S1a). Magnetic resonance imaging of the bilateral ankles showed multiple sites of tenosynovitis and synovitis. He was HLA-B27 negative and ANA positive 1:640. He had no clinical immunodeficiency, with an appropriate response to childhood immunizations and normal serum immunoglobulin levels (Additional file 1: Table S1).

**Fig. 1:**
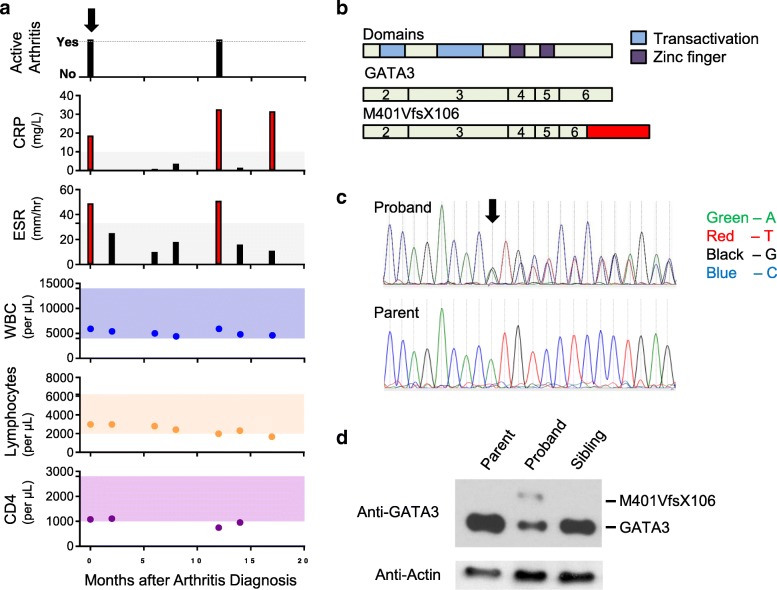
Autoimmune arthritis in proband with GATA3 mutation. **a** The proband clinical course from diagnosis of psoriatic JIA at time 0 to 17 months post diagnosis. Two episodes of clinical arthritis are black bars with the time of therapy initiation denoted by a downward arrow. The erythrocyte sedimentation rate (ESR) in millimeters per hour and C-reactive protein (CRP) in milligrams per liter are shown with abnormal values highlighted in red. Total white blood cells per microliter (WBC), absolute lymphocytes per microliter, and CD4+ cell values remain around the lower limits of normal. Normal lab values are denoted as shaded areas. **b** The functional transactivation and zinc finger domains of GATA3 aligned to GATA3 and the proband M401VfsX106. Exons are numbered. The mutant C-terminal extension is red. **c** Sequencing of complementary DNA in PBMCs from the proband and parent; the colors represent bases: green denotes A, red T, black G, and blue C. The arrow indicates the mutated residue. The proband has equal transcript of GATA3 and M401VfsX106 sequences. **d** Western blot analysis of GATA3 in PBMC from the parent, proband, and sibling showing GATA3 and M401VfsX106. Actin is a loading control

He was managed with intra-articular corticosteroid injections in bilateral knees and ankles that resulted in improved inflammation of both arthritis and psoriasis. He initiated maintenance therapy with methotrexate. He responded well to therapies, with a subsequent transient episode of ankle arthritis in the setting of an acute otitis media (Fig. 1a). He has low normal levels of total white blood cells, with absolute lymphocytes and CD4+ lymphocytes persistently around the lower limits of normal (Fig. 1a). Paralleling this, his CD3+, CD8+, and CD19+ lymphocytes have been around the lower limits of normal, with CD16&56+ lymphocytes overall normal (Additional file 1: Figure S1b). On therapy, he has had no unusual infections.

### Genetic studies

Trio WES on the proband and his parents revealed a de novo c.1201_1202delAT: pMet401ValfsX106 variant in exon 6 of the *GATA3* gene (Additional file 1: Figure S1c). Analysis under different inheritance models (i.e., autosomal de novo, homozygous recessive, compound heterozygous, X-linked de novo, X-linked recessive models) did not identify other reportable variants in the exome analysis. Our proband’s variant caused a frameshift starting with codon methionine 401, changing it to valine and creating a stop-loss at position 106 in the new reading frame (M401VfsX106). This caused 44 correct amino acids to be replaced by 105 incorrect amino acids (Fig. 1b). M401VfsX106 retained the domains important for GATA3 dimer formation and DNA binding. This variant has not been observed GnomAD (accessed March 22, 2019) nor reported in JIA or HDR patients.

### GATA3 expression

GATA3 RNA and protein expression were assessed in PBMC from the proband and family controls. The amplified proband cDNA exhibited expression of both GATA3 and M401VfsX106 at approximately equal transcript levels, indicating the mutant mRNA escapes nonsense-mediated decay (Fig. 1c). Protein analysis of PBMCs showed the proband expressed both GATA3 and M401VfsX106, whereas parent and sibling expressed only GATA3 (Fig. 1d). The proband expressed less GATA3 than controls, likely due to heterozygosity. The proband M401VfsX106 was expressed at a lower level than the proband GATA3.

### GATA3 transcriptional activity

**Fig. 2:**
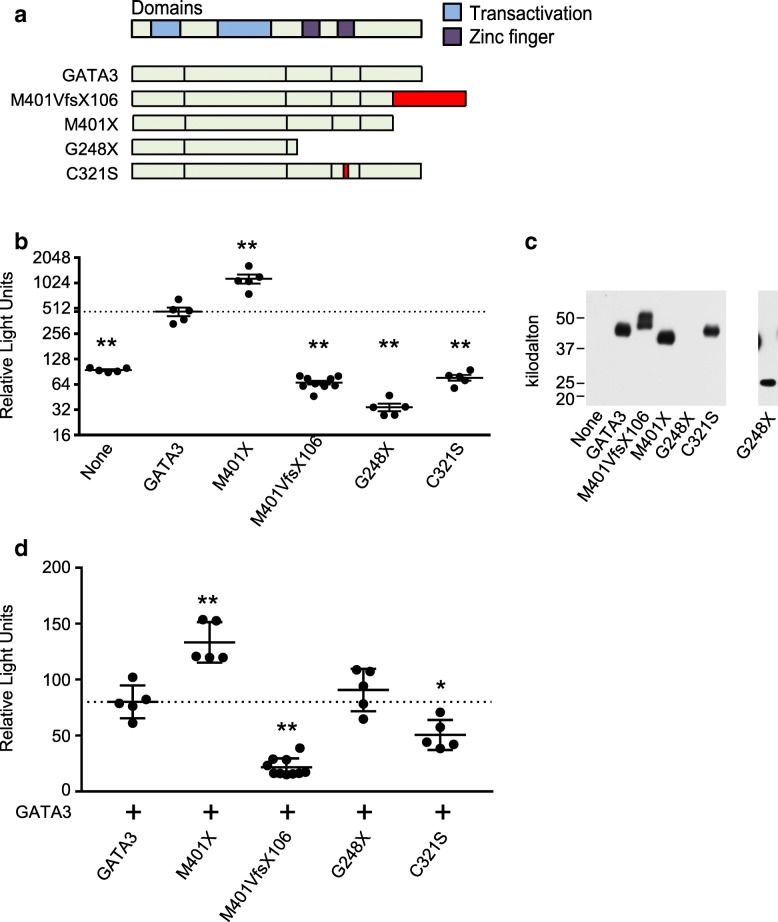
Mutant GATA3 transcriptional activity. **a** The functional transactivation and zinc finger domains of GATA3 aligned to wild type, M401X truncation, and the HDR-causing mutants M401VfsX106, G248X, and C321S. The mutant C-terminal extension and the missense mutation in C321S are red. **b** Luciferase activity in HeLa cells expressing a GATA promotor-driven luciferase vector and no construct, GATA3, a M401X truncation mutant, or the HDR-causing mutants M401VfsX106, G248X truncation, and C321S. Mean and SEM are shown. A statistically significant difference from GATA3 is indicated by *P < 0.05 and **P < 0.01. **c** Western blot analysis of GATA3 and mutants in HeLa cells. A longer exposure is shown for the detection of the G248X mutant. **d** Luciferase activity in HeLa cells co-expressing GATA3 and GATA3 or the mutants. Mean and SEM are shown. A statistically significant difference from GATA3 on the GATA3 background is indicated by *P < 0.05 and **P < 0.01

GATA3 function was measured by assessing the ability of mutant or wildtype protein to drive transcription of a GATA-luciferase reporter construct in HeLa cells, which do not express GATA3. Constructs included GATA3, M401X truncation, and the HDR-causing mutants M401VfsX106, G248X, and C321S (Fig. 2a). All constructs were expressed in HeLa cells at the predicted molecular weights, though G248X was expressed at a lower level (Fig. 2c). GATA3 stimulated a significant increase in luciferase expression in HeLa cells (Fig. 2b). The M401X truncation stimulated an even greater increase in luciferase expression, suggesting the natural C-terminus decreases downstream transcription. All HDR mutants had significantly decreased luciferase expression compared to GATA3, indicating the homodimers have reduced transcriptional activity.

Mutants were co-expressed with wildtype GATA3 to assess their impact on GATA3 transcriptional activity (Fig. 2d). Co-expression of GATA3 with M401X further increased luciferase expression. Co-expression with M401VfsX106 significantly reduced luciferase expression, indicating a dominant negative effect on GATA3 function. This is consistent with co-expression with C321S, which also has a dominant negative effect. Comparing the transcriptional activity of M401X and M401VfsX106, the proband C-terminal elongation clearly alters GATA3 function.

### Autoimmune disease-associated GATA3 mutant transcriptional activity

**Fig. 3:**
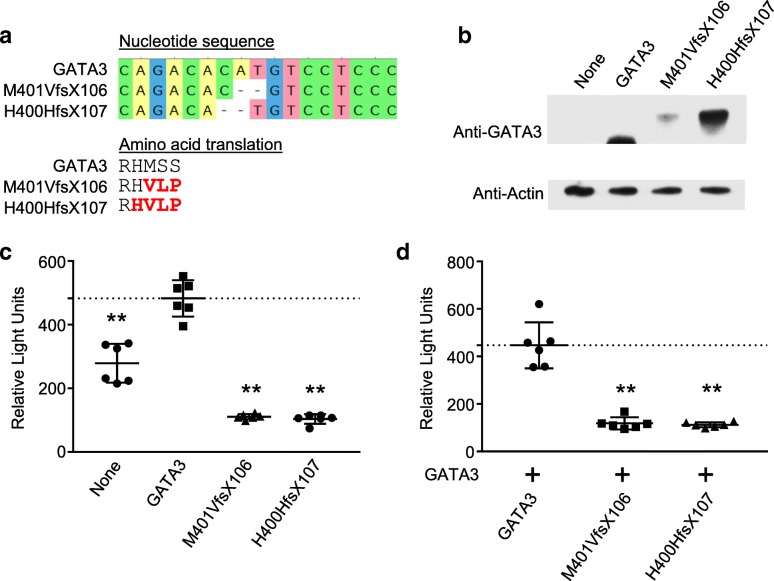
Two pediatric HDR patients with autoimmune disease have similar mutant GATA3. The proband (c.1201_1202delAT, p.M401VfsX106) and HDR patient with type I diabetes (c.1200_1201delCA, p.H400HfsX107) are compared. **a** Sanger sequencing results from plasmids containing the mutant constructs, M401VfsX106 and H400HfsX107, are shown aligned to the GATA3 sequence. Both mutations are a 2 nucleotide deletion. The amino acid translation from the selected sequence is shown. **b** Western blot analysis of GATA3, M401VfsX106, and H400HfsX107 in HeLa cells. **c** Luciferase activity in HeLa cells expressing a GATA promotor-driven luciferase vector and GATA3, M401VfsX106, and H400HfsX107. Mean and SEM are shown. A statistically significant difference from GATA3 is indicated by *P < 0.05 and **P < 0.01. **d** Luciferase activity in HeLa cells co-expressing GATA3 and GATA3, M401VfsX106, or H400HfsX107. Mean and SEM are shown. A statistically significant difference from GATA3 on the GATA3 background is indicated by *P < 0.05 and **P < 0.01

The autoimmune disease-associated HDR-causing mutants M401VfsX106 and H400HfsX107 are linked with JIA and type 1 diabetes. The respective nucleotide changes are c.1201_1202delAT and c.1200_1201delCA. Each mutation is a 2-nucleotide deletion that results in frame shift causing the same aberrant extended amino acid sequence (Fig. 3a). Both constructs were expressed in HeLa cells and have the same increased molecular weight as compared to GATA3 (Fig. 3b).

GATA3 function was measured by assessing the ability of mutant or wildtype protein to drive transcription of a GATA-luciferase reporter construct in HeLa cells. GATA3 stimulated a significant increase in luciferase expression in HeLa cells (Fig. 3c). Both M401VfsX106 and H400HfsX107 significantly decreased luciferase expression compared to GATA3, indicating the homodimers have reduced transcriptional activity (Fig. 3c). Constructs were co-expressed with wildtype GATA3 to assess the impact on GATA3 transcriptional activity. Both M401VfsX106 and H400HfsX107 result in significantly reduced luciferase expression, indicating both have a dominant negative effect on GATA3 function (Fig. 3d).

### GATA3 ex vivo functional activity in T helper cell differentiation

**Fig. 4:**
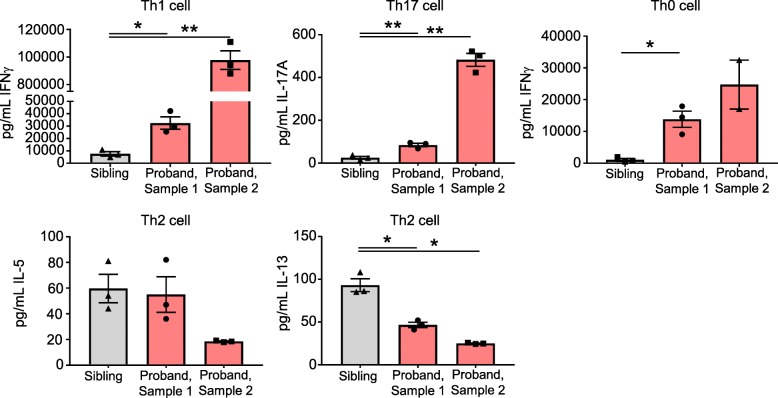
T helper cell differentiation and cytokine secretion. Ex vivo expression of cytokines from polarized Th1, Th2, Th17, and Th0 cell cultures. The expressed cytokines are IFNγ for Th1 cells, IL-5 and IL-13 for Th2 cells, and IL-17A for Th17 cells. IFNγ expression is shown from Th0 cells. PBMC cultures were prepared in biologic triplicate. Three PBMC collections are shown, with proband (sample 1) and sibling (collected 12 months after JIA diagnosis) and proband (sample 2) (collected 9 months after JIA diagnosis). Assays are *n* = 3 except proband (sample 2) Th0 is *n* = 2. Sibling and proband are compared. Mean and SEM are shown with *P < 0.05 and **P < 0.01

PBMCs were stimulated to differentiate along Th0, Th1, Th2, or Th17 cell pathways. Effector cells were re-stimulated and levels of secreted effector cytokines determined. Cytokines for the respective cell types include Th1: IFNγ, Th2: IL-5 and IL-13, Th17: IL-17A, and Th0: IFNγ (Fig. 4). The proband was compared to his similarly aged sibling, which is essential for minimizing age-related effects on the immune system. There are two timepoints of PBMC collection shown for the proband, indicating that the identified abnormalities are persistent over time. The proband had increased production of effector cytokines from the Th1 and Th17 pathways. The proband had no increase in IL-5 and a decrease in IL-13 production for effector cytokines from the Th2 pathway. In the proband, an undifferentiated Th0 cell produced IFNγ, which is a characteristic Th1 cytokine (Fig. 4).

## Discussion

We present the first known case of autoimmune arthritis associated with a mutation in *GATA3*. The proband M401VfsX106 protein is expressed and has a dominant negative function on GATA3 transcriptional activity. The proband PBMCs have markedly increased differentiation along the Th1 and Th17 pathways, with decreased differentiation along the Th2 pathway. Unexpectedly, Th0 cells from the proband express high levels of IFNγ like that of effector Th1 cells, supporting the notion that GATA3 is a major repressor of the Th1 differentiation pathway in this age group of children.

GATA3 is a transcription factor that functions as a homodimer, with each monomer containing transactivation domains, zinc finger domains, and a poorly understood C-terminus. HDR is caused by loss of GATA3 function. Most HDR-causing mutants disrupt the zinc finger domains, which are important for binding DNA [12, 13]. The M401VfsX106 mutation does not disrupt the zinc fingers, making the mechanism of action less clear. Two similar HDR-causing mutants have been identified. One frameshift occurs at amino acid 407 and adds a 98 residue C-terminal extension. Experimental assessment of this construct found that a homodimer had reduced protein expression and undetectable DNA binding [12]. The other frameshift occurs at amino acid 400 and adds a 107 residue extension (H400HfsX107) that is very similar to the proband, and this child developed type I diabetes [11]. We performed in vitro functional studies on this mutant showing that it produces the same mutant GATA3 with a dominant negative effect as the proband. It is remarkable that two HDR patients with similar mutations in GATA3 both developed autoimmune disease, which suggests a shared mechanism.

Similar to other HDR patients, the proband has decreased GATA3 function. His autoimmune disease could be due to the M401VfsX106 dominant negative mechanism and/or changes in the C-terminus. In breast cancer, somatic mutations in GATA3 are common with roughly half the reported mutations resulting in a C-terminal frameshift with stop-loss and extension [23]. A mechanism of aberrant epigenetic regulation in these mutations is supported by a frameshift at amino acid 409 that is selectively inhibited by specific histone methyltransferases [24] and a frameshift at amino acid 330 that results in decreased and increased expression of different genes [25]. The role of the proband C-terminal extension in epigenetic regulation could be clarified by assessing C-terminal truncation mutants in a stable cell line or overexpression system.

Autoimmune diseases are associated with increased Th1 and Th17 activity and increased IFNγ, which are recapitulated in the proband’s ex vivo Th cell differentiation assays. During Th cell differentiation, epigenetic changes occur at key genomic loci. In studies of the *IFNG* locus, epigenetic changes including gain and loss of histone modifications and DNA methylation occur during Th cell differentiation along the Th0, Th1, and Th2 lineages [26]. These epigenetic modifications are aberrant in the nonobese diabetic mouse model of insulin-dependent diabetes, resulting in Th0 cells that spontaneously express IFNγ [26–28]. Our proband has altered Th cell differentiation and produces Th0 cells that spontaneously secrete IFNγ, conceivably because of an aberrant epigenetic landscape. GATA3 is a key negative epigenetic regulator of Th1 differentiation [29] and loss of GATA3 may relieve this inhibition and allow IFNγ expression to proceed unopposed. Chromatin immunoprecipitation studies are ongoing to examine the epigenetic landscape at the *IFNG* locus to test this hypothesis.

JIA is divided into multiple subtypes [30]. Children with psoriatic JIA comprise two subpopulations, an early onset form similar to ANA-positive oligoarticular and polyarticular JIA and a later onset form similar to adult psoriatic arthritis [31, 32]. The characteristics of the proband JIA including the age of diagnosis, polyarticular, and ANA positive suggest that this child is similar to the ANA-positive polyarticular JIA subtype. In children with oligoarticular and polyarticular JIA, synovial fluid was found to contain an increase in T cells with a Th1 phenotype compared to the peripheral blood [6]. In enthesitis-related JIA, synovial fluid was found to contain an increase in Th1 and Th17 cells with a decrease in Th2 cells compared to the peripheral blood [7]. These studies focus on synovial fluid; however, the results parallel the proband Th cell differentiation abnormalities and suggest the presence of this Th cell pattern in JIA.

*GATA3* has been assessed as part of a deep whole genome sequencing study of children with polyarticular JIA [33]. In this study, *GATA3* contained no statistically significant enrichment of known single nucleotide polymorphisms (SNPs), novel SNPs, known insertions and deletions, and novel insertions and deletions [33]. The function of GATA3, including expression level and genomic binding, has not been characterized in Th cells from children with JIA. Our study supports GATA3 function as a potential contributing factor for autoimmunity in JIA and provides a monogenetic mutation that can be used as a tool to better understand this process.

A limitation of our study is that we have identified a single HDR patient that developed JIA. We have approached this by assessing the proband at multiple timepoints and performing in vitro studies in additional HDR-causing mutations. The identification of a second HDR patient with autoimmune disease with a similar mutation in *GATA3* further supports the connection between *GATA3* and autoimmune disease. We have assessed in vitro GATA3 function in a luciferase expression system. An alternative would be to use chromatin binding to determine genomic GATA3 binding sites. These assays are complicated by GATA3 functioning as a dimer, with the clinically relevant cells likely expressing both mutant GATA3 and GATA3. Our approach has been to use proband PBMCs to perform ex vivo differentiation of Th cells and assess effector cytokine secretion. It is possible that other cell types and processes are altered by the mutant GATA3, and experiments assessing these cells would need to be performed. The Th cell is a relevant cell for JIA and autoimmune arthritis as supported by studies highlighted in this discussion.

Our study shows that decreased and altered GATA3 activity coincides with autoimmune arthritis and suggests that GATA3 function is important in JIA. Mice that overexpress GATA3 are protected from joint inflammation in a model of arthritis [34]. Further, a therapeutic has been developed to target reduction of GATA3 for atopic disease [35]. Similarly, a strategy to induce GATA3 may be a potential therapeutic approach for patients with autoimmune arthritis.

## Conclusions

Our research expands the phenotypic spectrum for *GATA3* mutations and presents a possible genetic cause for this patient’s arthritis. The presented data indicate the proband expresses mutant GATA3 with a dominant negative function on transcriptional activity. T cells are altered, with markedly enhanced differentiation along the Th1 and Th17 pathways and decreased differentiation along the Th2 pathway. These results provide new insight into GATA3 function and suggest that decreased or altered GATA3 activity coincides with autoimmune arthritis.

## Additional file

Additional file 1: **Figure S1.** Autoimmune arthritis in the proband**. a** The pedigree for HDR disease, proband clinical diagnoses, and criteria met for psoriatic juvenile idiopathic arthritis (JIA). **b** The proband clinical course from diagnosis of psoriatic JIA at time 0 to 17 months post diagnosis. Absolute CD3+, CD8+, CD19+, and CD16&56+ cells per μL values are shown. Normal lab values are denoted as shaded areas. **c** The functional transactivation (TA) and zinc finger (ZF) domains of GATA3 aligned to wild type and the proband c.1201_1202delAT. The amino acids in the C-terminus in wild type versus the proband C-terminal extension are shown. **Table S1.** The proband has no clinical immunodeficiency. He has normal serum immunoglobulin levels and an appropriate response to childhood immunizations. (DOCX 251 kb)

## Data Availability

The datasets supporting the conclusions of this article are included within the article. Please contact the corresponding author for data requests.

## Abbreviations

cDNA: Complementary DNA
ELISA: Enzyme-linked immunosorbent assay
GATA3: GATA binding protein 3
HDR: Hypoparathyroidism, sensorineural deafness, and renal disease
IFN: Interferon
IL: Interleukin
JIA: Juvenile idiopathic arthritis
PBMCs: Peripheral blood mononuclear cells
PCR: Polymerase chain reaction
RA: Rheumatoid arthritis
Th: T helper
WES: Whole exome sequencing
