# Supplementary material for: Juvenile idiopathic arthritis associated with a mutation in GATA3
Source: Arthritis Res Ther. 2019 Jun 25;21:156. doi: 10.1186/s13075-019-1946-3 (PMC6593533; doi:10.1186/s13075-019-1946-3)
Supplement: Supplementary file 1 — Figure S1. Autoimmune arthritis in the proband. a The pedigree for HDR disease, proband clinical diagnoses, and criteria met for psoriatic juvenile idiopathic arthritis (JIA). b The proband clinical course from diagnosis of psoriatic JIA at time 0 to 17 months post diagnosis. Absolute CD3+, CD8+, CD19+, and CD16&56+ cells per μL values are shown. Normal lab values are denoted as shaded areas. c The functional transactivation (TA) and zinc finger (ZF) domains of GATA3 aligned to wild type and the proband c.1201_1202delAT. The amino acids in the C-terminus in wild type versus the proband C-terminal extension are shown. Table S1. The proband has no clinical immunodeficiency. He has normal serum immunoglobulin levels and an appropriate response to childhood immunizations. (DOCX 251 kb) [file 13075_2019_1946_MOESM1_ESM.docx]

**Fig. S1** Autoimmune arthritis in the proband**. a** The pedigree for HDR disease, proband clinical diagnoses, and criteria met for psoriatic juvenile idiopathic arthritis (JIA). **b** The proband clinical course from diagnosis of psoriatic JIA at time 0 to 17 months post diagnosis. Absolute CD3+, CD8+, CD19+, and CD16&56+ cells per μL values are shown. Normal lab values are denoted as shaded areas. **c** The functional transactivation (TA) and zinc finger (ZF) domains of GATA3 aligned to wild type and the proband c.1201_1202delAT. The amino acids in the C-terminus in wild type versus the proband C-terminal extension are shown.


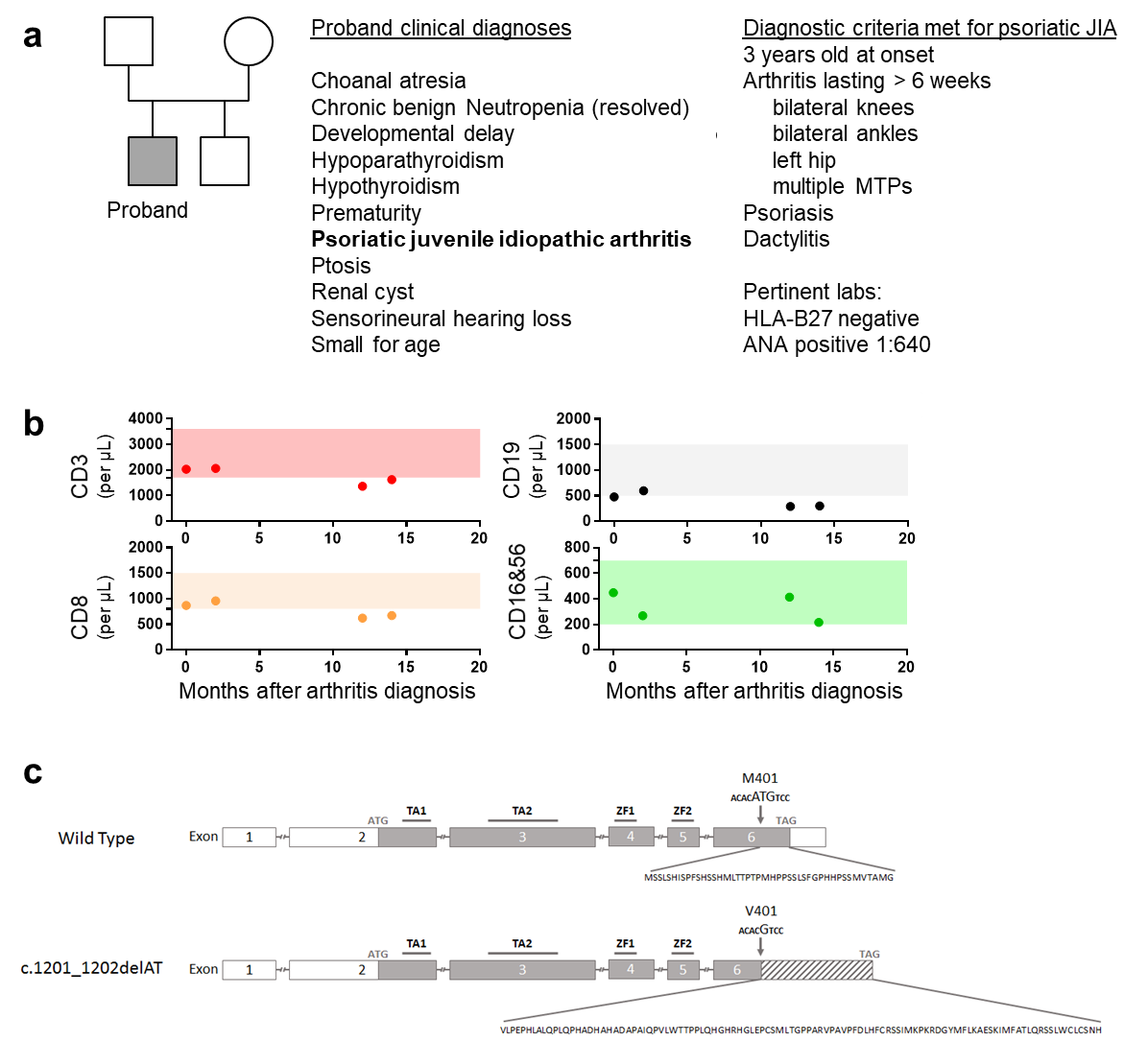


**Table S1** The proband has no clinical immunodeficiency**.**  He has normal serum immunoglobulin levels and an appropriate response to childhood immunizations.

**
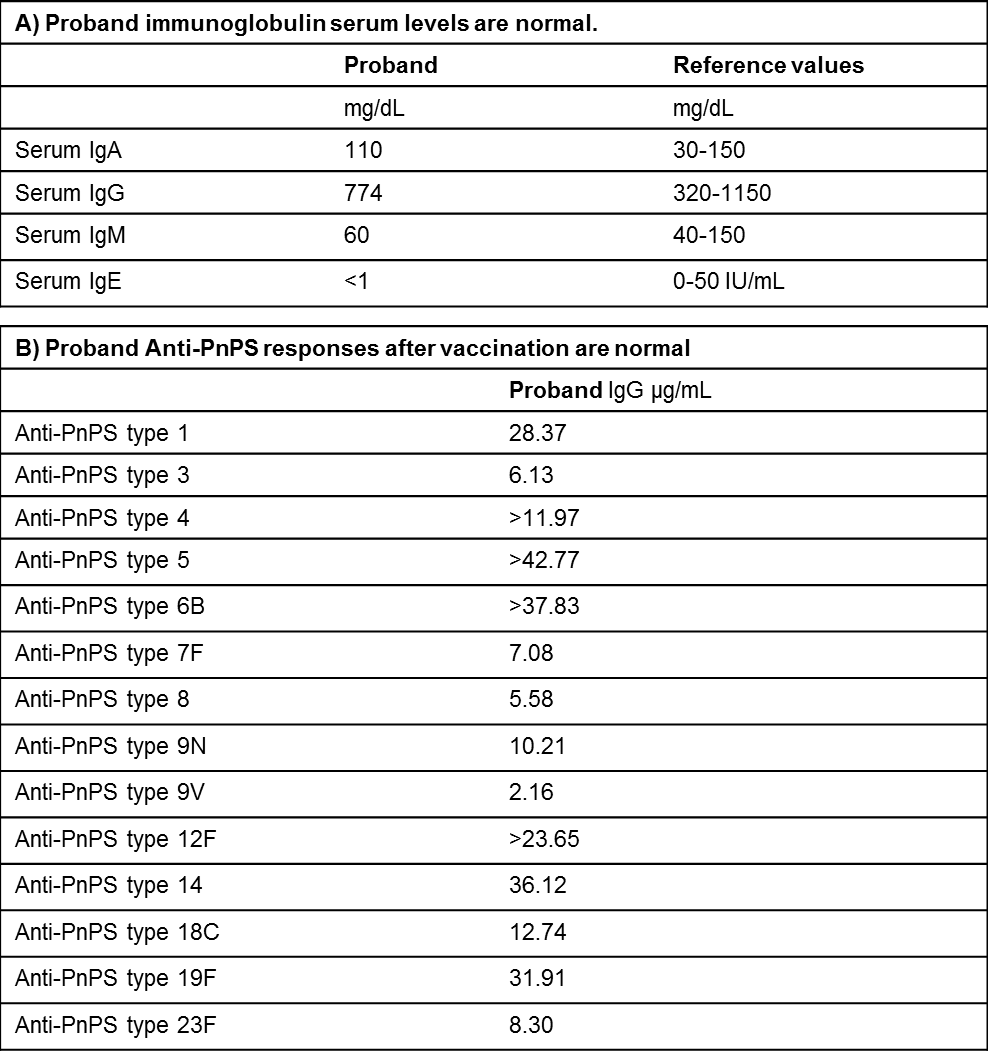
**
